# The Anti-vivisection Agitation

**Published:** 1898-11

**Authors:** E. V. Wilcox

**Affiliations:** Professor of Biology, Montana State College


THE JOURNAL
OF
COMPARATIVE MEDICINE AND
VETERINARY ARCHIVES.

VOL. XIX. *NOVEMBER, 1898.* No. 11.

### THE ANTI-VIVISECTION AGITATION.

BY E. V. WILCOX, PH.D.,
PROFESSOR OF BIOLOGY, MONTANA STATE COLLEGE.

ANYONE who has taken the trouble to familiarize himself with the progress of scientific thought and investigation during the past two centuries can scarcely have failed to notice the persistent opposition which has been set up to every new method of research and every new triumph. The announcement of a scientific discovery has invariably been met with protests and cries of indignation from the strongholds of fanaticism. As is well known, the conflict has always been unequal and the victory on the side of science. But ignorance and prejudice "spring eternal" in the human mind, and as soon as one conflict is settled the war breaks out afresh in some other field. Scientific workers have had to accustom themselves to all sorts of opprobrious epithets until such men have come to expect, as punishment for having made any real contribution to the sum of human knowledge, the regular volley of abuse and curses.

In a country with such perfect freedom of speech as we enjoy in the United States, it is certainly an inalienable right of the, unfortunately, large number of people so disposed, to call other people by vile and offensive names. Indulgence in abusive language, however, is, after all, a quite harmless occupation for the class of persons who enjoy that sort of pastime. No scientific worker need feel in the least hurt at being called "wretch," "criminal," "fiend," "hell-fiend," etc., as is quite the fashion in a certain class of sensational literature which is being circulated broadcast over the country. To the average sensation-monger such words may sound very formidable. But these and other similar words have been in the mouths and in the writings of the opponents of science from time immemorial, and the ordinary observer has a right to demand that these people use some new tactics in their invectives, for the sake of variety if for no other reason. Aside from all considerations of the etiquette of honorable controversy, we would humbly suggest the abandonment of this method of attack, for the simple reason that it has never had any effect except to make its users despicable in the sight of all well-behaved persons.

Vivisection is, perhaps, one of the latest scientific methods of research to be attacked by the enemy. In this country the attack has been carried on more or less assiduously for the past thirty years. The controversy has been an extremely unequal one, in so far as one side has been unceasingly talking and agitating, and the other side has only occasionally made any protest. Scientists, as a rule, have great faith in the ultimate reaction of common sense against the absurd misrepresentations of the anti-vivisectionists, and therefore seldom take the trouble of giving an explanation of the purpose of their work to people who persistently misinterpret these explanations. In the hundreds of controversies in geology, physics, chemistry, philology, anthropology, and biology the agitators against scientific investigations have simply been allowed to talk until finally they came to a realizing sense of the inanity of their own words. When will that day come for the vivisection question?

The anti-vivisectionists compare quite unfavorably with former anti-scientific agitators. Heretofore the enemies of science have taken the trouble to acquaint themselves after a fashion with the meaning of the scientific tendency which they opposed. No one can accuse the anti-vivisectionists of studying the questions which they so glibly discuss. As Prof. Hodge says:[1] "In several hundred anti-vivisection publications I am unable to find a passage which reveals the least conception on the part of their writers of the real purpose which a physiologist has in his work." Not only do they not understand the purpose of the physiologist, but they even fail to distinguish between physiology and anatomy.

### *The Case of the Anti-vivisectionists.*

We now proceed to examine the case of the anti-vivisectionists from some of their recent publications. We shall give quotations

[1] Popular Science Monthly, vol. xlix. p. 614.

from these writings which will illustrate their main characteristics, viz:

1. Dense ignorance of the meaning and bearings of the whole question.

2. Utter disregard of facts.

3. Intense pleasure in abusive language toward scientists.

4. Morbid delight in the sensational.

Anti-vivisection writings, as a rule, have the intellectual value and moral quality of a highly sensational newspaper account.

"The construction of human or animal bodies can be learned by books and dissections; and an authority no less than Dr. Michener, who ought to know, if anyone, declares that he has found the students who obtained their knowledge in this legitimate way quite as expert as those who resorted to degrading and revolting cruelty."

Quite true; but no one dissects living animals in order to learn the "construction" of the body. Experiments are conducted upon living animals to learn something of their physiology, not of their anatomy. The sentence above quoted asserts, also, by implication, a distinct falsehood, viz.: That the vivisector actually does dissect living animals for the purpose of learning their anatomy. The one sentence is, therefore, a good index of the ignorance of the author on the subject of vivisection and of her rhetorical trickery.

Anti-vivisectionists set great store by the quotations which they are able to offer from the physicians and surgeons who are opposed to vivisection. Now, physiology is a recent science as compared with anatomy, and it is a well-known fact that there are many medical men who still seem to think there is really no such science as physiology, and that all we know about medicine comes from the dissection of dead human bodies and from "experiments on human patients." It is, perhaps, needless to say that the opinions of such men in physiological experiments are utterly valueless. We are told that "indeed, no discussion regarding this momentous question can be conclusive which does not accord place to the opinions thus deliberately formed by the eminent authorities whom we are privileged to quote."

Dr. Charles Bell Taylor is then quoted as saying that "the anæsthetic properties of ether and chloroform were discovered by experiments upon human patients, not by vivisection of animals." Unfortunately for Dr. Taylor, this does not involve a matter of opinion, but a question of fact, and the facts are against the gentleman. The facts are that animals were anæsthetized and operated upon in that condition before anæsthetics were used on man. It would repay the gentleman to look up the records of the Massachusetts General Hospital on this point.

In regard to the discovery of the circulation of the blood, "the foremost abdominal surgeon of England" is quoted as follows: "And it seems to me, as a skilled anatomist, that the circulation of the blood could not be either discovered or demonstrated by any other method than the use of the injecting syringe, the microscope, and the dead body."

Such nonsense is almost beneath criticism. If the learned professor could demonstrate the circulation of the blood in a dead body he would thereby demonstrate that the "dead body" was alive. How in the name of common sense could we "discover" the circulation of the blood by forcing an injecting mass through the bloodvessels? All that this witness or any other person could demonstrate in the dead body is the presence of tubes. These tubes, the arteries and veins, were known and described long before the circulation of the blood was discovered. But that knowledge never helped us to a discovery of the movement of the blood.

Many other equally false and nonsensical statements are quoted from this man who, the anti-vivisectionists say, is "the foremost abdominal surgeon in England." For example, this great surgeon is quoted as saying:

"As regards the discovery of the circulation of the blood by Harvey, my answer to the question whether he made any substantial contribution to his success by his experiments on living animals is obtained from his own writings, and I say that he made no such contribution." What are we to judge from such a statement? Has the professor not read the works of Harvey? Or is it simply a prejudiced misstatement? Perhaps it will help to decide this matter if the reader is informed that the witness just quoted is a man who has repeatedly been shown to be utterly unreliable and untrustworthy in his testimony on this subject. Aside from his misrepresentations, his nauseating drivel on the subject of vivisection would be unworthy of notice if it were not continually thrown in our face by the anti-vivisectionists as medical science. Anyone who will merely take the trouble to read the titles of the chapters of Harvey's work, *De Motu Cordis et Sanguinis in Animalibus*, will find that Harvey depended upon vivisection at every step in his investigation.

In one of their circulars the anti-vivisectionists have quoted from Gordon as follows: "No sufficient analogies exist in the animal kingdom from which to draw useful conclusions." This quotation includes only a part of a sentence, a very important qualifying phrase being for some reason omitted. But even with the qualification added, the sentence shows such a profound ignorance of the progress of comparative anatomy that the witness certainly cannot pose as giving expert testimony on the subject. For a case like this there is only one effective remedy to prescribe, and that is to take a course in comparative surgery and comparative physiology.

We may be pardoned for adding on this point a quotation from a certain Dr. Blackwood: "Carried out as these brutal acts are on animals whose physiological construction and life-habits bear no possible relation to humankind, it is impossible that other than false doctrine should be the result of their cruel and degrading work." Here the phrase "physiological construction" is another example of the confusing of anatomy and physiology, a common mistake among the anti-vivisectionists. The essential physiological processes in all animals, from amœba to man, are the same. There are, of course, certain idiosyncrasies, but these scarcely differ more in individuals of different species than in those of the same species. For anyone to assert, as the last witness does, that these processes in the lower animals "bear no possible relationship to humankind" is simply to insult the intelligence of the average reader. Perhaps the witness knows of some animals in which the digestive functions are performed by the brain, and in which the kidneys aërate the blood. Or is the occult witness talking of astral animals?

We may add that it is only necessary to turn at random to any page of anti-vivisection literature to find everywhere therein overwhelming evidence of profound ignorance of the whole question at issue. But ignorance is by no means the greatest defect of the anti-vivisectionists.

Let us now pass to a consideration of another characteristic of their writings, namely, disregard of facts.

Concerning vivisection, a certain Dr. Foster says:

"It is contrary to the best methods of hygiene, sanitation, and dietetics and the proper study of therapeutics and materia medica."

Every unprejudiced historian of the development of these branches of medical science will at once protest that this a strange misrepresentation of the facts. The best and only scientific methods of hygiene, sanitation, and dietetics, in so far as they have any reasonable foundation, are based entirely on results derived from vivisection. The anti-vivisectionists have adopted the rhetorical tricks of all charlatans, patent-medicine venders, and quack doctors. They deliberately distort the facts on the subject of vivisection and then raise a great hue and cry about the public being "hoodwinked" by the friends of science.

We quote again from an anti-vivisectionist circular:

*Extract from Report Showing Existence of Abuses.*

510 FIFTH STREET, N. W., WASHINGTON, D. C., April 15, 1896.

HON. J. H. GALLINGER, United States Senate:

It was my lot for a number of years to be engaged in the microscopical division of the Army Medical Museum, and I saw practised the most inhuman and barbarous mutilations of the dumb animals under the supervision and with the sanction of the United States officers in charge. A desired part or section of the animal would be removed, not under anæsthesia, and the poor beast would be then placed back in its cage or vessel until it suited the convenience of the operator to help himself to another portion, so long as the animal would survive these tortures. I have thus seen animals with eyes, section of brain, and other parts removed, and kept in reserve for future experiments for a number of days, and all for the verification and repetition of results obtained and published years ago.

"These unnecessary horrors, practised openly with the sanction of United States medical officers, make me think that stringent laws are needed to restrict such proceedings. None should be permitted not calculated to give additional useful information, and then under perfect anæsthesia and under the supervision of a board of competent men assigned to that duty.

"Very respectfully, your obedient servant,

"L. E. RAUTERBERG, M.D."

I have reproduced the letter of Dr. Rauterberg entire, so as not to appear to criticise him from fragments of his statement. This letter purports to be written as a protest against existing abuses, and contains the usual quantity of sensational matter. But when we come to inquire as to who this witness is and with what authority he speaks, we find that his testimony is not worth the paper on which it is written. As the present Secretary of Agriculture has shown in a circular published in May, 1897, we have here to deal with another case of misrepresentation. The only statement of the whole letter which will stand criticism is that the witness "was engaged in the microscopical division of the Army Medical Museum." The records show, moreover, that Dr. Rauterberg was "engaged" in a clerical capacity, but that he has not been engaged in any capacity in that institution for the past twenty-three years. The witness, however, and the anti-vivisectionists who quote him, attempt to give the impression that his statement is an authoritative account of the "existence of abuses." Thus we get an idea of the trustworthiness of the witness. Perhaps the witness will be able to justify himself *à la* anti-vivisection mode for omitting dates, and thus foisting a mere rhetorical trick upon the public as serious argument. But we must set him another task. We would respectfully request him to substantiate the sensational portion of his letter. Secretary Wilson is authority for the statement that "responsible medical officers connected with the museum testify that there have been no painful experiments performed there since 1870, nor, so far as they are informed, previous to that time."

Thus we have in this witness a man who was never connected with the institution which he reviles, except in the position of a clerk, and that twenty-three years ago, who is quite irresponsible, but who has the audacity to give out false impressions by a vague statement and to allow his imagination to run riot while he abuses responsible officers of the institution.[1]

We read in an anti-vivisectionist circular concerning vivisection that "there is nothing in the world about which more falsehood is told." This undoubtedly contains a truth which we are now in a position to appreciate.

We shall now examine a witness who is apparently one of the main pillars of the whole anti-vivisection movement. This man is Philip G. Peabody, A.M., M.D., LL.D., Boston, Mass., President of the New England Anti-vivisection Society, President of the National Constitutional Liberty League, President of the National Scientific Family Culture Institute, Vice-President of the Massachusetts Society for the Prevention of Cruelty to Animals, Vice-President of the Illinois Anti-vivisection Society. Dr. Peabody certainly has a keen scent for the sensational, and he is possessed of an ability in writing this sort of literature which would

[1] The anti-vivisectionists, in publishing Dr. Rauterberg's letter, have been careful to omit the first and most important sentence of the original. The omitted sentence reads as follows: "I have promised a lady, very much interested in the bill relating to vivisection now under consideration by your committee, to address you on the subject in a few brief words." Who is responsible for the absurd letter, the "lady" or Dr. Rauterberg?

admit him at once to the columns of the illustrated Sunday edition of a modern yellow newspaper. None of the witnesses heretofore examined have considered it necessary to take oath to the truthfulness of their statements. Dr. Peabody, however, seems to have had some slight misgivings as to the limits of human credulity. At any rate, he went before a justice of the peace in New Hampshire and swore that his account was true. This absurd proceeding in itself is enough to make one suspicious of the witness' story. And in the general affairs of life no one would believe a witness under oath who was not otherwise trustworthy.

Other anti-vivisectionists have had excellent success in working up sensational tales with regard to various American colleges and medical schools. But our present witness must go abroad for his sensational material. The masterpiece of this anti-vivisectionist was to be based on an alleged investigation of the methods of vivisection in the Imperial Veterinary School at Alfort, France. Dr. Peabody's methods of investigation are extremely interesting. To get evidence on the question as to whether or not anæsthetics were used he interrogated "the highly intelligent *attaché* whose express duty it is to show visitors about and give them information." The quotation is from Peabody's own statement under oath. The witness does not speak of meeting or of attempting to find any responsible officers of the school from whom to get reliable information; but, as if ashamed of the mission on which he was bent, he was content with bringing home "revelations" from a janitor. Unfortunately, there is in this country a large audience for exciting scandals. But no one can claim to have the best interests of the rising generation at heart who would deliberately set out to scrape together sensational matter on foreign soil, and publish it broadcast in our land to poison the minds of the youth. Anti-vivisectionists have much to answer for, and, perhaps, for nothing more serious than for the degrading and demoralizing influence of their literature.

We quote once more from Dr. Peabody: "I have for years past personally witnessed an immense amount of vivisection of all kinds from the least to the most terrible, and of many kinds of animals from the horse to the guinea-pig. I have never yet in all my experience seen any anæsthetics in, or about, or in use in any laboratory, with one exception. I have a personal acquaintance with many vivisectors, including a number of the best known ones in this country and Europe. I have hundreds of times conversed with them. No vivisector has claimed, or pretended, to use any anæsthetic in conversation with me.[1] They have always freely admitted that they never used them."

Before criticising this statement we will quote for comparison the testimony of Prof. C. F. Hodge: "During over ten years' active experience in three laboratories in this country and a number of the leading laboratories abroad I have never had occasion to perform or witness an experiment of this painful class. Discovery of new anæsthetics and more recent methods of operation have doubtless reduced the pain of experimentation even below Yeo's estimate. In all laboratories in this country, and equally abroad, I have found anæsthetics adequately and uniformly employed."

Now let us compare these two statements. On the one side we have the testimony of a practical physiologist, an honest investigator, who has contributed to the progress of experimental physiology. Prof. Hodge is, moreover, a responsible gentleman with an official position and under obligation to know whereof he speaks. On the other side we find a mere collector of sensations, a man who visits laboratories for the purpose of adding to his collection, and who is possessed of the morbid desire of being shocked by imagined cruelties, so morbid, indeed, that he would feel disappointed if he should be convinced of the non-existence of his imagined horrors.

I was a student at Harvard College from 1891 to 1895, and am therefore somewhat acquainted with the work which was done at that institution during the years mentioned. I saw animals vivisected under the influence of anæsthetics. I never saw any vivisected without the use of anæsthetics. Numerous student friends in the Harvard Medical School told me that vivisection was never performed there without anæsthetics. Did Dr. Peabody ever visit Harvard Medical School? Again, in consequence of the sensational stories and malicious exaggerations of the anti-vivisectionists, a bill was presented to the Massachusetts Legislature asking to have persons appointed as inspectors of vivisectional experiments. This happened in 1896. In the winter of 1896 a similar bill was presented to the National Congress. Both these bills were, of course, defeated, not, as the anti-vivisectionists say, "by those who wished neither inspection nor restriction," but simply by the inability of the petitioners to present any case. As Prof. Hodge says for Massachusetts: "None of the petitioners for the bill were able to cite a single case or the reasonable suspicion of a case of

1 We can only guess at the meaning of this sentence. It seems to mean that the vivisectors actually endured Dr. Peabody's conversation without taking ether.

abuse of vivisection.'' The same thing was true of the bill in Congress. The only pretended instance of the abuse of the practice in the District of Columbia was the aforementioned imbecile letter of a man who had been a clerk in the Army Medical Museum twenty-three years ago.

Now, will Dr. Peabody please enlighten us as to what he was doing during these legislative discussions of vivisection? Here was an excellent opportunity to meet vivisectionists who habitually use anæsthetics. Not only were the anti-vivisectionists unable to find a case where anæsthetics were not used, but the vivisectors offered abundant proof that anæsthetics are continually used in their experiments. The testimony of Prof. Hodge, moreover, is a flat contradiction of Dr. Peabody's statement in every particular. Prof. Hodge's testimony is based on an incomparably more intimate and extensive knowledge of the subject than is that of Dr. Peabody. These facts, therefore, brand Dr. Peabody's account as insincere, and present to him the following dilemma: Either his statements are misrepresentations, or he has made no honest effort to get the facts of the case.

(To be continued.)

